# Evaluation of classical and novel autoantibodies for the diagnosis of Primary Biliary Cholangitis-Autoimmune Hepatitis Overlap Syndrome (PBC-AIH OS)

**DOI:** 10.1371/journal.pone.0193960

**Published:** 2018-03-19

**Authors:** Henry H. Nguyen, Abdel Aziz Shaheen, Natalia Baeza, Ellina Lytvyak, Stefan J. Urbanski, Andrew L. Mason, Gary L. Norman, Marvin J. Fritzler, Mark G. Swain

**Affiliations:** 1 University of Calgary Division of Gastroenterology and Hepatology. Hospital Dr NW, Calgary, Alberta Canada; 2 University of Calgary Department of Medicine. Hospital Drive NW Calgary, Alberta, Canada; 3 University of Alberta Division of Gastroenterology. Zeidler Ledcor Centre Edmonton, Alberta Canada; 4 University of Calgary Department of Pathology & Laboratory Medicine. Calgary, Alberta Canada; 5 Inova Diagnostics. San Diego, CA, United States of America; Texas A&M University, UNITED STATES

## Abstract

**Background and aims:**

Up to 20% of Primary Biliary Cholangitis (PBC) patients are estimated to have features that overlap with Autoimmune Hepatitis (AIH). Patients with PBC-AIH overlap syndrome (PBC-AIH OS) have been reported to exhibit suboptimal responses to ursodeoxycholic acid therapy, and are more likely to progress to cirrhosis. Anti-double stranded DNA (anti-dsDNA) and anti-p53 have been previously suggested to be potential autoantibodies for identifying patients with PBC-AIH OS. In our well defined PBC patient cohorts, a comprehensive assessment of various classical and novel autoantibodies was evaluated for their utility in identifying PBC-AIH OS patients.

**Methods:**

PBC-AIH OS was classified according to the Paris criteria and PBC as per the European Association for the Study of the Liver guidelines. Biobanked serum samples from 197 patients at the University of Calgary Liver Unit and the University of Alberta were analyzed for classical and novel autoantibodies. Anti-dsDNA was measured by the *Crithidia luciliae* immunofluorescence (CLIFT) assay (1:20 dilution) and chemiluminescence (CIA: QUANTA Flash®, Inova Diagnostics, San Diego). Anti-p53, anti-Ro52/TRIM21, anti-YB 1, anti-GW182, anti-Ge-1, and anti-Ago 2 were measured by either an addressable laser bead immunoassay (ALBIA) or line immunoassay (LIA). Autoantibodies against MIT3, gp210, sp100, LKM1, SLA, and the novel autoantibodies Hexokinase-1 (HK-1), and Kelch like protein 12 (KLHL-12) were measured using QUANTA Lite® ELISA assays. We applied non-parametric methods to compare the biomarkers frequencies between study groups. We used multivariate adjusted models and AUROC to compare the diagnostic accuracy of the different autoantibodies alone or in combination with serum biochemistry.

**Results:**

16 out of 197 PBC patients (8.1%) were classified as PBC-AIH OS. Compared to PBC patients, PBC-AIH OS patients were similar in age (median: 59 vs. 63, P = 0.21) and female predominance (94% vs. 89%, P = 1.00). Anti-dsDNA-by CLIFT (37.5% in PBC-AIH OS vs 9.9% in PBC alone, P <0.01) was the only autoantibody associated with PBC-AIH OS; a finding consistent with previous reports. Significant elevation in serum ALT (62 IU/L in PBC-AIH OS vs 37 IU/L in PBC alone, P < 0.01), and serum IgG (17.6 g/L in OS vs 12.1 g/L in PBC alone, P <0.01) were observed in patients with PBC-AIH OS receiving medical/immunosuppressive therapy. In a multivariate model, positive anti-dsDNA by CLIFT, ALT and IgG were significant predictors of PBC-AIH OS with an area under the receiver operator curve (AUROC) value of 0.84.

**Conclusions:**

Consistent with previous findings, the presence of anti-dsDNA by CLIFT is associated with PBC-AIH OS. Contrary to previous reports, anti-p53 was not associated with PBC-AIH OS. Our comprehensive evaluation of various classical and novel autoantibody biomarkers including Ro52/TRIM21, anti-p53, anti-KLHL-12 and anti-HK-1 were not significantly associated with PBC-AIH OS. Our findings highlight the ongoing need for the research and development of new autoantibody biomarkers to aid in the diagnosis of PBC-AIH OS.

## Introduction

Autoimmune liver diseases (ALDs) such as primary biliary cholangitis (PBC) and autoimmune hepatitis (AIH) are distinct clinical entities characterized by specific biochemical, histological, and immuno-serological profiles. The diagnosis of PBC is supported by the presence of elevated cholestatic liver enzymes, positive anti-mitochondrial antibodies (AMA), and lymphocytic infiltration/granulomatous destruction of interlobular bile ducts on liver biopsy. AIH is characterized by the presence of elevated hepatocellular liver enzymes, positive anti-smooth muscle antibodies, or elevated immunoglobulin G, and features of piecemeal necrosis/interface hepatitis on liver biopsy. Although these diagnostic criteria allow for the classification of PBC and AIH as distinct entities, there is a subset of patients (up to 20% of PBC) who harbor overlapping features of both PBC and AIH, a clinical subset referred to as PBC-AIH overlap syndrome (PBC-AIH OS) [1]. The implications for a diagnosis of PBC-AIH OS, having both important prognostic and treatment implications, extends beyond disease classification. Specifically, higher rates of complications associated with end stage liver disease (esophageal varices, GI bleeding, ascites, and liver transplant) and lower rates of 5-year survival were reported in PBC-AIH OS patients [2,3]. Furthermore, the response to conventional therapy with ursodeoxycholic acid (UDCA) differs substantially for patients with PBC as compared to PBC-AIH OS. For example, UDCA treatment has been shown to be insufficient for disease control in patients with PBC-AIH OS [4]. Therefore, the use of combination therapy involving both UDCA and immunosuppressant therapy (i.e. corticosteroids) has been shown to result in more favourable biochemical and histological responses in patients with PBC-AIH OS compared to monotherapy with either agent alone [2,4–6]. Given this difference in treatment regimens and overall health outcomes in patients with PBC-AIH OS, early identification and appropriate medical intervention is important for preventing progressive liver disease. However, a significant remaining challenge is the accurate diagnosis of PBC-AIH OS that currently relies on a combination of serological, biochemical and histopathological features of both PBC and AIH [7]. Autoantibodies has been previously suggested to be a useful non-invasive marker for diagnosing patients with PBC-AIH OS. Specifically, both anti-double stranded DNA (anti-dsDNA) and anti-p53 antibodies have been previously reported to be significantly associated with PBC-AIH OS, compared to PBC alone [8,9]. Given this the aim of our study was to perform a comprehensive examination of bio-banked sera from well-defined PBC and biopsy proven PBC-AIH OS patients for various conventional and novel autoantibodies to improve our ability to identify patients with PBC-AIH OS in an accurate and timely manner. Using multivariate analysis, the presence of specific autoantibodies alone or in combination with liver biochemistry results were assessed for their utility to identify patients with PBC-AIH OS.

## Methods

### Study design

All adult patients with a diagnosis of PBC alone or PBC-AIH OS were followed by the Hepatology groups at either the University of Calgary Liver Unit (UCLU, Calgary Canada) or University of Alberta (Edmonton, Canada). The serum from these patients were collected and biobanked at -80°C spanning a 10-year period from 2007 to 2017. Serum biochemistry was evaluated at the time of diagnosis and up to one-year after autoantibody testing. This time frame was chosen as the evaluation of liver biochemistry was not uniformly carried out for all patients at fixed time points and was dependent on the practice of the individual Hepatologist. The diagnosis of PBC was made in accordance with international practice guidelines requiring two of the following criteria: 1) cholestatic liver biochemistry, 2) AMA-positivity and/or 3) compatible liver histopathology [7,10]. The diagnosis of PBC-AIH OS was made in patients fulfilling two of three criteria for both PBC (as mentioned above) and AIH. The diagnostic criteria for AIH include two out of the following three: 1) Elevated ALT >5 times upper limit of normal 2) Elevated IgG > 2 times upper limit of normal, or positive anti-smooth muscle antibody positivity and/or 3) compatible liver biopsies. To ensure accurate classification of PBC-AIH OS, all patients had a liver biopsy, which was reviewed by a blinded expert pathologist. The research was approved by the University of Calgary Conjoint Health Research Ethics Board (CHREB) and conducted in accord with the Helsinki Declaration. Informed consent for use of serum and medical records was acquired from patients prior to the research work being done. Patient clinical and biochemical data was obtained via retrospective chart review. Biochemical parameters including serum alkaline phosphatase (AP), alanine aminotransferase (ALT), aspartate aminotransferase (AST), total bilirubin, gamma glutamyl transferase (GGT), and immunoglobulin G (IgG) were recorded. All patient information was anonymized prior to being analyzed.

### Autoantibody testing

Aliquots of the biobanked PBC or PBC-AIH OS serum samples were processed and tested by Mitogen Advanced Diagnostics Laboratory (Calgary, AB, Canada) for various novel autoantibodies associated with autoimmune liver disease. Anti-double stranded DNA (anti-dsDNA) was assessed using the *Crithidia luciliae* immunofluorescence (CLIFT) assay (1:20 dilution; Inova Diagnostics, Inc., San Diego, CA, USA) and a chemiluminescence immunoassay (CIA: QUANTAFlash, Inova Diagnostics). Antibodies against tumor suppressor gene p53 (anti-P53), tripartite motif protein 21 (anti-Ro52/TRIM21), Y-box protein 1 (anti-YB 1), mRNA processing bodies/GW bodies (anti-GW182), Ge-1 (anti-Ge-1), and Argonaute protein (anti-Ago 2) were measured by an addressable laser bead immunoassay (ALBIA) on a Luminex 100 flow fluorometer (Luminex Corp., Austin TX, USA)

Antibodies against liver-kidney microsomal antibody (anti-LKM), triple hybrid fusion MIT3 antigen (anti-MIT3), soluble liver antigen (anti-SLA), nuclear antigen (anti-sp100), nuclear envelope protein (anti-gp210) were evaluated using commercially available and US FDA-cleared QUANTALite ELISA Assays (Inova Diagnostics). Antibodies against Hexokinase 1 (anti-HK-1) and Kelch-like protein 12 (anti-KLHL 12) were measured by QUANTA Lite ELISA (Research use only, Inova Diagnostics).

Patient demographics were described using median averages. Frequency of autoantibodies was compared between PBC alone and PBC-AIH OS groups using non-parametric statistical methods. The performance of patient serum biochemistry and autoantibody profiles in predicting PBC-AIH OS was determined using multivariate analysis and area under receiver operating curve (AUROC). A P-value less than 0.05 was considered statistically significant.

## Results

Serum from 197 patients with either PBC alone or PBC-AIH OS were evaluated in our study in which 8.1% (16/197) were diagnosed with PBC-AIH OS. Patient demographics, autoantibody profiles, and serum biochemistry in PBC-alone and PBC-AIH OS patient groups are summarized in Table 1. 93.8% of PBC-AIH OS were female as compared to 89.0% in PBC-alone, (P = 1.00) and the median age (58.5 yrs in PBC-AIH OS vs 63.0 yrs PBC alone; P = 0.21) were similar between the two groups.

**Table 1:** Comparison of clinical characteristics (demographics and liver biochemistry) in patients with primary biliary Cholangitis-Autoimmune hepatitis overlap syndrome and primary biliary cholangitis alone.

Characteristics	PBC-AIH OS (n = 16)	PBC alone (n = 181)	P-value
**Age, median years**	58.5	63	0.25
**Female Gender, %**	93.8	89	1.00
**Alanine Aminotransferase (IU/L)**	62	37	0.01*
**Immunoglobulin G (g/L)**	17.6	12.1	0.01*
**Total Bilirubin (μmol/L)**	12	10	0.10
**Alkaline Phosphatase (IU/L)**	153	163	0.51

Age is expressed as median years. Proportion of female gender and liver biochemistry is expressed as a mean average.

* A p-value less than 0.05 is considered statistically significant

Characteristics of individual patients diagnosed with PBC-AIH OS are summarized in Table 2. Most PBC-AIH OS patients (11/16) had received immunosuppression as part of their medical therapy at the time of serum collection for biochemistry and autoantibody testing. Evaluation of the various autoantibodies highlighted a significantly greater frequency of CLIFT anti-dsDNA in PBC-AIH OS compared to PBC alone (37.5% vs 9.9% respectively, P = 0.006).

**Table 2:** Clinical variables, serum biochemistry and autoantibody markers in all patients diagnosed with primary biliary Cholangitis-Autoimmune hepatitis overlap syndrome evaluated in the study.

Patients	Age & Sex	ALT	IgG	Anti-dsDNA	Medical Therapy
**1**	63 (F)	63	11.2	Positive	Prednisone + Ursodiol
**2**	57 (F)	36	ND	Negative	None
**3**	73 (F)	37	16	Negative	None
**4**	76 (F)	22	ND	Negative	None
**5**	68 (F)	32	ND	Negative	Ursodiol
**6**	56 (F)	71	11.5	Negative	None
**7**	43 (F)	60	27.9	Positive	None
**8**	63 (F)	30	16.2	Positive	Prednisone and MP
**9**	52 (F)	608	24	Negative	None
**10**	38 (F)	529	17.6	Negative	None
**11**	58 (F)	43	14.1	Positive	Prednisone + Ursodiol
**12**	53 (F)	87	8.2	Negative	Ursodiol
**13**	62 (F)	1522	18.7	Positive	None
**14**	59 (F)	40	23.2	Positive	None
**15**	53 (F)	142	30	Positive	None
**16**	73 (F)	444	18.3	Negative	Prednisone

Clinical variables included patient age, sex and medical therapy at the time of autoantibody and serum biochemistry testing. Serum ALT and immunoglobulin G are expressed in units of IU/L and g/L respectively. Anti-dsDNA was evaluated using the *Crithidia luciliae* immunofluorescence (CLIFT) assay (1:20 dilution).ALT; Alanine Aminotransferase, F; female, Anti-dsDNA; anti-double stranded Deoxyribonucleic acid, IgG; Immunoglobulin G, MP; Mercaptopurine

Of note, none of the CLIFT positive sera were positive for anti-dsDNA by CIA. The frequencies of the remainder of the autoantibodies, including anti-Ro52/TRIM21, anti-p53, anti-KLHL 12, anti-HK 1, anti-GW182, anti-YB1, anti-Ge1, anti-Ago2, anti MIT3, anti-LKM, anti-SLA, anti-sp100 and anti gp210 were similar between PBC-AIH OS and PBC alone groups (Table 3). Serum biochemistry including AP, AST, total bilirubin, and GGT were not significantly different between PBC-AIH OS and PBC-alone groups. In contrast, significantly elevated serum ALT (median: 62.0 in PBC-AIH OS vs 37 in PBC alone; P = 0.01) and serum IgG (median: 17.6 in PBC-AIH OS vs 12.1 in PBC alone; P = 0.01) were observed in patients with PBC-AIH OS; the majority of whom were receiving medical therapy/immunosuppressants.

**Table 3:** Comparisons of frequencies of autoantibodies in patients with primary biliary cholangitis autoimmune hepatitis overlap syndrome (PBC-AIH OS) and patients with Primary Biliary Cholangitis (PBC) alone.

Autoantibodies	PBC-AIH OS (n = 16)	PBC alone (n = 181)	P-value
**Anti dsDNA-Crithidia assay (%)**	37.5	9.9	0.006*
**Ro52/TRIM21 (%)**	25	17.7	0.50
**Anti-p53 (%)**	31.3	34.8	1.00
**Anti-YB-1 (%)**	31.3	37.6	0.79
**Anti-GW-182 (%)**	43.8	37.0	0.60
**Anti-Ge 1 (%)**	31.3	22.7	0.54
**Anti-Ago 2 (%)**	37.5	31.5	0.59
**Anti-MIT 3 (%)**	73.3	87.3	0.13
**Anti-LKM (%)**	6.7	0	0.08
**Anti-SLA (%)**	13.3	2.2	0.07
**Anti-Hexokinase 1 (%)**	40.0	43.7	1.00
**Anti-SP100 (%)**	40.0	28.7	0.38
**Anti-GP210 (%)**	40.0	20.4	0.10
**Anti-Kelch Like Protein 12 (%)**	40.0	22.7	0.20

Anti-double stranded DNA (anti-dsDNA) was assessed using the Crithidia luciliae immunofluorescence (CLIFT) assay and a chemiluminescence immunoassay. Antibodies against tumor suppressor gene p53 (anti-P53), tripartite motif protein 21 (anti-Ro52/TRIM21), Y-box protein 1 (anti-YB 1), mRNA processing bodies/GW bodies (anti-GW182), Ge-1 (anti-Ge-1), and Argonaute protein (anti-Ago 2) were measured by an addressable laser bead immunoassay (ALBIA) on a Luminex 100 flow fluorometer. Antibodies against liver-kidney microsomal antibody (anti-LKM), triple hybrid fusion MIT3 antigen (anti-MIT3), soluble liver antigen (anti-SLA), hexokinase 1 (anti-HK-1), nuclear antigen (anti-sp100), nuclear envelope protein (anti-gp210) and Kelch-like protein 12 (anti-KL12) were evaluated using the commercially available QUANTA Lite Enzyme Linked Immunosorbent Assays.

* A p-value less than 0.05 was considered statistically significantly.

**Fig 1:**
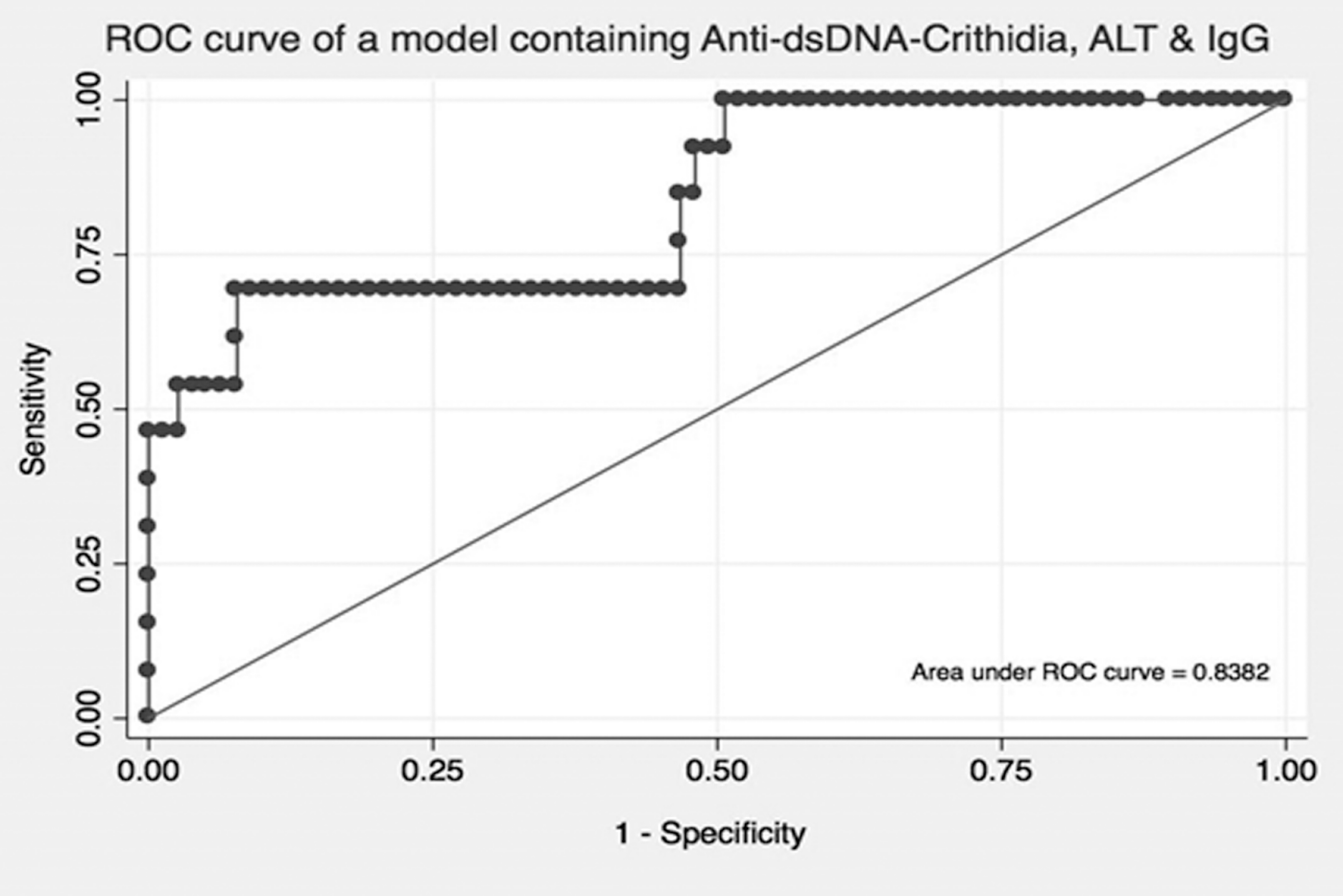
Receiver Operating Characteristics (ROC) curve utilizing the combination of serum ALT, IgG and presence of *Crithidia luciliae* assayed anti-dsDNA to identify patients with primary biliary cholangitis autoimmune hepatitis overlap syndrome. Serum ALT and immunoglobulin G are expressed in units of IU/L and g/L respectively. Anti-dsDNA was evaluated using the *Crithidia luciliae* immunofluorescence (CLIFT) assay (1:20 dilution). Utilizing all three variables resulted in the highest AUROC value of 0.84.

In univariate analysis, the presence of CLIFT anti-dsDNA, elevated ALT and IgG levels (while on medical/immune therapy) resulted in respective odds ratios of 5.43 (95% CI [1.77, 16.7]), 1.01 (95% CI [1.00, 1.01]) and 1.11 (95% CI [1.02, 1.21]) (Table 4). Multivariate analysis models utilizing different combinations of anti-dsDNA, ALT, and IgG and the respective area under the receiver operating curve (AUROC) are shown in Table 5. Anti-dsDNA in combination with either ALT or IgG alone resulted in a AUROC value of 0.75 and 0.74 respectively. A model utilizing only ALT in combination IgG yielded a AUROC value of 0.78. Utilizing all three variables in multivariate analysis resulted in the highest AUROC value of 0.84 for predicting patients with PBC-AIH OS (Fig 1).

**Table 4:** The odds ratios of serum biochemistry and an autoantibody marker significantly associated with patients classified as having primary biliary cholangitis autoimmune hepatitis overlap syndrome.

Variables	Odds Ratio	AUROC
**Anti-dsDNA-Crithidia assay**	5.43	0.64
**Alanine Aminotransferase**	1.01	0.70
**Immunoglobulin G**	1.11	0.72

Serum ALT and immunoglobulin G are expressed in units of IU/L and g/L respectively. Anti-dsDNA was evaluated using the *Crithidia luciliae* immunofluorescence (CLIFT) assay (1:20 dilution). Area under the Receiver Operating Characteristic (AUROC) values are shown for each individual variable. ALT; Alanine Aminotransferase, Anti-dsDNA; anti-double stranded Deoxyribonucleic acid, IgG; Immunoglobulin G

**Table 5:** Area under the receiver operating characteristic curve (AUROC) values for combinations of variables significantly associated with primary biliary cholangitis autoimmune hepatitis overlap syndrome.

Variables	AUROC
**Anti-dsDNA-Crithidia + ALT + IgG**	0.84
**Anti-dsDNA-Crithidia + ALT**	0.75
**Anti-dsDNA-Crithidia + IgG**	0.74
**IgG + ALT**	0.78

Serum ALT and immunoglobulin G are expressed in units of IU/L and g/L respectively. Anti-dsDNA was evaluated using the *Crithidia luciliae* immunofluorescence (CLIFT) assay (1:20 dilution). ALT; Alanine Aminotransferase, Anti-dsDNA; anti-double stranded Deoxyribonucleic acid, IgG; Immunoglobulin G

## Discussion

The diagnosis of PBC-AIH OS is estimated to occur in up to ~20% of patients with PBC, but in most studies the prevalence is typically <10% [1]. The diagnosis itself has important implications for overall liver health, prognosis and decision making regarding optimal medical management [3,5,6]. Currently, the diagnosis requires a constellation of serum biochemistry and histological findings suggestive of both PBC and AIH. Non-invasive testing with autoantibody markers have been previously suggested to be useful in identifying patients with PBC-AIH OS. In our patient cohort we evaluated a wide array of classic and novel autoantibodies that have either been associated with PBC, and/or general autoimmune liver disease, along with serum biochemistry to identify patients with PBC-AIH OS. The frequency of PBC-AIH OS in our cohort was 8%, which is within previous reported ranges of 4.8 to 9.2% [4,11,12]. Our PBC and PBC AIH OS cohorts evaluated 181 PBC-alone patients, and 16 patients with PBC-AIH OS. In comparison to previous studies of PBC-AIH OS, our study evaluated one of the largest disease cohorts assessed to date.

The presence of positive CLIFT anti-dsDNA autoantibody was significantly associated with PBC-AIH OS. In our cohort, the frequency of CLIFT anti-dsDNA was significantly (~ 4-fold) higher in PBC-AIH-OS (40% PBC-AIH OS vs 10% in PBC alone). Anti-dsDNA as a biomarker of PBC-AIH OS in our cohort is consistent with a previous study wherein a significant difference in the frequency of anti-dsDNA was reported (60% in PBC-AIH OS vs 4% in those with PBC alone) [8]. The difference in absolute proportion of patients with anti-dsDNA positive PBC-AIH OS between our study and the Italian report may be due to differences in referral patterns and the patient populations studied. Of note, anti-dsDNA antibodies were not confirmed by CIA, an assay that uses a synthetic dsDNA polynucleotide and has high sensitivity and specificity for SLE, as well as high concordance with CLIFT in studies of SLE sera [13]. Our observations suggest that a unique dsDNA epitope is targeted by PBC-AIH OS antibodies or that there are unique targets in *Crithidia* kinetoplast that are targeted by PBC-AIH OS. The CLIFT, which uses the hemoflagellate *Crithidia luciliae* as a substrate, was developed in 1975 by Lars Aarden and colleagues [14,15], and is still widely used for the detection of anti-dsDNA, particularly in the setting of diagnosing systemic lupus erythematosus [16]. Although it is thought that the kinetoplast is composed of highly compacted dsDNA in the absence of histones and hence is an ‘ideal’ anti-dsDNA target, more recent evidence points to a highly complex representation of dsDNA attended by histone-like proteins and other proteins involved in regulating the expression of kinetoplast DNA [17–19]. Nevertheless, with some exceptions, the CLIFT is renowned for high specificity and positive predictive value for SLE and is the reason why it is often used for confirmation of a positive result obtained with another method. Since the kinetoplast is a modified mitochondrion, the finding of kinetoplast-reactive antibodies in PBC-AIH OS is intriguing and may point to other mitochondria targets that are not seen in PBC alone. One novel mitochondrial target is HK-I [20], but in our study, antibodies to HK-1 were not significantly associated with PBC-AIH OS. Hence, further studies are required to precisely identify the CLIFT target reacting with PBC-AIH OS antibodies.

Abnormalities in the p53 tumor suppressor gene have often been associated with solid tumors [21] and anti-p53 antibodies have also been associated with AIH [22]. In a Japanese study, anti-p53 was detected in 50% of patients with PBC-AIH OS as contrasted to only 2.4% in sera from PBC alone and hence was suggested to be useful for the diagnosis of PBC-AIH OS [9]. Contrary to these findings, significant differences in frequencies of anti-p53 were not identified in our PBC AIH OS and PBC alone patient groups. Discrepancies in the association of anti-p53 and PBC-AIH OS may potentially be attributed to differences in the composition of patient populations studied (i.e. Japanese vs Canadian populations), or the smaller cohort of patients evaluated in the Japanese study (n = 41 PBC alone and n = 8 with PBC-AIH OS). Furthermore, it was not clear whether the Japanese study required liver biopsy confirmation for all patients classified as PBC AIH OS; therefore potentially overestimating the number of patients with PBC-AIH OS [8].

Anti-SLA and anti-LKM target a transfer ribonucleoprotein and a cytochrome p450 enzyme respectively [23–26]. Both autoantibodies have been described in patients with AIH [26,27]. In our study, both autoantibodies trended towards increased frequency in the overlap group vs PBC alone, but were not statistically significant. Autoantibodies targeting microRNA Processing Bodies (PB/GW bodies) have been described broadly in various autoimmune conditions and have been suggested as being potentially complementary biomarkers in PBC patients [28,29]; these include GW182, Ge-1 and Ago2 [28–31]. However, the frequency of PB/GWB autoantibodies was similar in the PBC-AIH OS and PBC alone cohorts. The presence of autoantibodies against nuclear protein gp210 in patients with PBC has been previously suggested to be associated with a worse prognosis [32–35]. Interestingly, one study found that patients harboring anti-gp210 antibodies were more likely to have features of interface hepatitis and lobular inflammation on liver biopsy [34]; findings suggestive of possible PBC-AIH OS. Moreover, another study found significantly higher frequencies of anti-gp210 in patients with PBC-AIH OS vs PBC alone [36]. However, we did not detect significantly higher frequencies of anti-gp210 in the serum of patients with PBC-AIH OS vs PBC alone. The differences in our findings for anti-gp210 in PBC-AIH OS may be potentially attributed to differences in patient populations (previous reports consisted of predominately patients of Japanese descent), smaller numbers of patients included in previous reports, and differences in assaying techniques.

Both anti-KLHL 12 and anti-HK 1 have been described as being highly specific markers for PBC that can be used in conjunction with other biomarkers, including anti-gp210 and anti-sp100 [20]. Interestingly, there is emerging evidence suggesting that anti-HK1 may be a marker of poor prognosis in PBC patients, with increased risk in liver disease progression and lower transplant free survival [37]. In our study, we found similar frequencies of anti-KLHL 12 and anti-HK 1 antibodies in patients with PBC-AIH OS and those with PBC alone.

Other factors that should be considered and further studied is whether treatment with immunosuppression can alter the titre/detection of autoantibodies in patients with PBC-AIH OS, and whether this has implications for prognosis/disease progression. In our cohort, 6/16 PBC-AIH OS patients were on concurrent immunosuppression in addition to UDCA when study serum was obtained. Data from previous studies have shown that titres of certain autoantibodies, including anti-gp210, ANA, AMA, ASMA, may change in response to immunosuppressive therapy [38]. As these patients were being treated with immunosuppressive medications, this could potentially have suppressed PBC-AIH OS-related changes in autoantibody levels. To date, no study has evaluated the effects of immunosuppression on the various PBC and/or AIH associated autoantibody titres evaluated in our study. Furthermore, whether these changes in autoantibody titres affect overall disease activity and prognosis remains unanswered. Previous studies evaluating changes in autoantibody titres often do not find an association with underlying disease activity [35]. It would be interesting for future studies in PBC-AIH OS to evaluate chronological changes in various autoantibody titres in response to therapy. However, this would be challenging as it would require a prospective approach in a relatively rare condition.

Our multivariate analysis highlighted the combination of anti-dsDNA in conjunction with elevated serum ALT and IgG was able to differentiate patients with PBC-AIH OS from those with PBC alone with a AUROC value of 0.84. The presence of elevated ALT is suggestive of underlying hepatocellular injury and IgG is an indicator of predominant plasma cell infiltration of the liver; both characteristic of AIH [39,40]. Our findings of elevated ALT as a predictor of PBC-AIH OS are congruent with findings from a recent study [41]. However, unlike this study, we did not identify AP, AST, or total bilirubin as being predictive of PBC-AIH OS. This difference in outcome may be related to the patient population studied, timing of serum collection relative to treatment and the higher total number of patients evaluated in our study. It is important to note that the magnitude of elevated median serum ALT and IgG observed in PBC-AIH OS patients were mild ranging ~1.5-2X above normal reference values. This may be in part due to 6/16 PBC-AIH OS patients having already received medical therapy / immunosuppressants at the time of serum testing; attenuating the overall degree of measurable liver injury in these patients. As outlined in the methods section, the retrospective nature of our study and the variability in practice amongst the different Hepatologists/care centers is the reason why some patients were already on therapy at the time of testing and why a 1-year inclusion window between autoantibody testing and liver biochemistry testing was chosen. Interestingly, when patients with PBC-AIH OS were evaluated, 15/16 had liver biochemistry and autoantibody testing done concurrently. In contrast, the timing between autoantibody and liver biochemistry testing in our PBC-alone group was variable. Overall, the statistically significant finding of a persistent mild elevation in ALT and IgG in PBC-AIH OS vs PBC-alone despite some patients being on medical therapy highlights the difference in underlying liver pathology in patients with PBC-AIH OS vs PBC-alone.

Our study, has some limitations that warrant discussion. The patient cohorts we studied, although well-defined for PBC-AIH OS and PBC alone, were retrospectively evaluated and may, therefore, represent an inherent selection bias. In addition, we studied 16 PBC-AIH OS patients, therefore calculations of autoantibody associations with clinical overlap disease may be underpowered. This limitation in power may mask potential positive associations between autoantibodies and PBC-AIH OS is not unique to our study and reflects PBC-AIH OS being a rare and an uncommonly diagnosed condition. To circumvent this, future studies will need to rely on collaborative efforts (i.e. Multi-center) and pooling of patient data/samples to increase the n-value and overall Statistical power.

## Conclusion

In conclusion, we performed a comprehensive assessment of a wide range of autoantibody biomarkers that have commonly been associated with autoimmune liver disease, in addition to serum biochemistry, to better identify patients with PBC-AIH OS. Our findings support previous reports of anti-dsDNA as a reliable marker of PBC-AIH OS. More importantly, our negative findings point to the ongoing need of autoantibody/biomarker development to aid in the diagnosis of patients with PBC-AIH OS. The presence of a combination of anti-dsDNA, elevated ALT and elevated IgG levels (despite therapy) should prompt a clinician to a potential diagnosis of PBC-AIH OS.
